# A Course of Clinical Lectures, Delivered at the Hôtel-Dieu, Paris, for the Session 1842-’43

**Published:** 1843-04-01

**Authors:** A. F. Chomel

**Affiliations:** Paris


### THE MEDICAL EXAMINER,

### And Retrospect of the Medical Sciences.

Vol. VI.] PHILADELPHIA, SATURDAY, APRIL 1, 1843. [No. 6.

### A COURSE OF CLINICAL LECTURES,

*Delivered at the Hôtel Dieu, Paris, for the Session* 1842–'43.

BY A. F. CHOMEL, M. D.

### LECTURE IV.

CASE I.—*Puerperal metro-peritonitis three days after delivery; complicated with severe pectoral disease. Death—Autopsy.*

At No. 3 of the Salle Saint-Bernard, commenced Dr. Chomel, is a woman who is attacked with puerperal metritis. The disease first appeared at the Maternity Hospital, where the woman was delivered. She tells us that, having suffered a good deal of distress from her position in life, her pregnancy was accompanied from the commencement by a *malaise*, nearly constant; and that when it had reached the seventh month, she suffered so much that she determined on entering the Maternity, where she was soon afterwards delivered. The labour, it appears, was natural, but afterwards there was a very abundant flow of blood, and subsequently severe pain in the hypogastrium.

On the third day after delivery she had a chill, the breasts swelled, and the abdomen became painful. Leeches were applied to the abdomen, which diminished somewhat the intensity of the pain; she had, however, general *malaise* until the seventh day, when she left the hospital and went home in a carriage, which seems to have shaken her a good deal. On reaching her house she was taken with a chill, and with pains in the limbs and lower part of the abdomen. She then determined on entering the Hôtel Dieu, where we found her condition as follows:

At first sight of this patient, there was an air of suffering and oppression. The belly was voluminous and meteoric; touching it gently produced pain, especially when you pressed on the hypogastric region. For two days previously she had had repeated bilious vomiting, with small alvine evacuations. On examining her *per vaginam*, we found the os tincæ soft, and a little sensible; but on introducing the end of the finger into the neck of the uterus, and endeavouring to displace that organ, there was anormal immobility, which seemed due to adhesions, which the fundus has contracted with the adjacent organs. On pressing with the hand on the hypogastric region, whilst the finger of the other hand was in the neck of the uterus, some obscure motions were determined, and we found that the fundus was several inches above the level of the pubis. The finger, on being withdrawn from the vulva, was bathed by a whitish, sticky liquid, somewhat fetid.

This collection of symptoms did not permit us to doubt, for a moment, the existence of a metro-peritonitis, in which the peritoneum was secondarily affected, which most frequently happens; the peritoneum ordinarily inflames after the uterus.

As general symptoms, we had great dryness of the tongue and mouth, with thirst. The skin was hot, the pulse one hundred and forty, and the features very much changed.

This affection differs materially from that which we call post-puerperal metro-peritonitis; it is, as a general rule, a much more severe disease. In our patient, happily, it appeared several days after her delivery; the initial chill occurred on the third day. Now, the more remote the period of the chill is from the day of delivery, the less severe is the disease which supervenes. The post-puerperal metro-peritonitis is, in this respect, like those phlegmasiæ which are produced by exterior causes, and which are always less grave than those which succeed to some general spontaneous cause; whilst puerperal metritis is consecutive to some profound general alteration of the system, having for a principal phenomena one or more chills, and is always a very grave disease. There is always fear of an inflammation, either of the veins, or of the lymphatics; and in either case the disease is serious. We have the more to fear a disease of this nature in our patient, from the fact that an epidemic of this kind is at present reigning at the Maternity, where she was taken sick. Hence our prognosis yesterday was unfavourable, although there were some encouraging symptoms.

To-day her condition has not improved. Her pulse is very frequent, (one hundred and sixty,) and very small. Her features are very much drawn, and her face has an earthy tint, which is always a bad omen. The discharges from the vulva are becoming intolerably fetid. They have the odour characteristic of metritis, where great alteration in the organ has occurred. On examining her this morning, I discovered on the anterior face of the vagina a tumour, with distinct fluctuation, which at first led to the suspicion of a purulent collection; but on introducing a catheter into the bladder, a large quantity of urine escaped; and on reëxamining her, the tumour had disappeared. There was here, therefore, a state of atony of the bladder, inducing retention of urine. It is, besides, a common symptom in women recently delivered; and you should, therefore, always attend to the state of the bladder.

In the course of yesterday she complained of pain in the right side of the chest. On percussion it was found flat; on auscultating her we discovered the respiration obscure below, and superiorly a bellows sound, with bronchial respiration. Here, then, we have a pleuritic effusion, complicating metro-peritonitis. It might happen that the liver, swollen, and pressed upwards, causes the dulness at the inferior portion of the right side. But at the superior portion of the lung respiration is obscure, and otherwise slightly anormal. We must, therefore, admit absolutely a lesion, not only of the pleura, but of the parenchyma of the lung itself. These symptoms, of course, render the prognosis more grave.

A large blister has been applied over the whole anterior surface of the right side of the chest, in the hope of making a favourable revulsion, both for the disease of the abdomen as well as that of the chest. At the same time we prescribed emollient injections, with the addition of the chloride of lime, into the vagina, with the view of correcting, as much as possible, the fetid nature of the discharges. Mercurial frictions on the abdomen, and purgative enemata, constitute the remainder of the treatment.

[Three days subsequently the patient succumbed. At the autopsy, there was found in the uterus a portion of placenta, with several coagula, surrounded by a sanious fluid, extremely fetid. The size and consistence of the uterus were nearly natural; its walls were in the condition we usually find them in on the eleventh or twelfth day after delivery. The uterine veins contained neither pus nor blood. In the broad ligament only there was a cavity of the size of an almond, filled with purulent matter. The peritoneum was covered with false membranes. In the peritoneal cavity there was nearly a pint of pus. There were false membranes in the pleura, especially about the summit of the lung, as well as upon the convex surface of the liver.]

CASE II.—*Organic affection of the heart—Hypertrophy of the ventricle, with valvular deficiency—General bronchitis—Abdominal tumour caused by the liver—Ascites, and infiltration of the lower extremities, and of the abdominal parietes, following protracted instrumental labour.*

In the same ward is a woman whose case is of great interest. When my attention was first directed to her she was almost in a state of suffocation, such was the extraordinary difficulty of respiration. Her face was swollen, her lips violet, and her lower extremities and abdomen œdematous. When she entered the hospital the preceding evening, the degree of suffocation was such, that a small bleeding was immediately practised. Questioned with regard to the origin and history of her present disease, we could learn only this. She had been lately delivered. (I shall refer to this point, which is important, again presently.) She at first told us that she had always been healthy before her present attack, which occurred about six weeks previously; on being, however, questioned more closely, she finally acknowledged that since the age of fifteen she had been subject to shortness of breath; that she also had laboured under palpitations, and became oppressed whenever she fatigued herself. She told us, besides, that she recollects, since her childhood, when playing with her comrades, that her breathing often became oppressed. And six weeks ago these symptoms became so much aggravated as to oblige her to obtain medical assistance.

This woman is the mother of five children, and does not appear, at least from what she tells us, to have experienced the ordinary effects which successive pregnancies generally produce on the organism. Her last delivery only was tedious, and was instrumental.

The actual condition of the patient led us to suspect the existence of an organic affection of the heart. We asked her if she had ever had an attack of rheumatism, and she answered us in the negative, and persisted, to our reiterated questions, in saying that she knew very well what rheumatism was, but that she had never suffered from it. Here is a negative fact to add to a vast number of positive facts of the same kind that we have collected, and which oppose the doctrine, now so prevalent relative to the coincidence between rheumatism and organic disease of the heart, and which demonstrates how cautious we should be when we draw general conclusions from certain isolated facts. The urine gave no evidence of renal disease. We then directed our attention to the central organ of the circulation. On percussing the præcordial region, we found dulness extending over a surface of nearly four square inches, inducing suspicion of an augmentation, in volume, of the heart. Auscultation was somewhat impeded by a blowing respiration, a veritable, general, sonorous rhonchus; so that it is with difficulty, and not without some precautions, that you can hear the sounds of the heart. But, in auscultating with attention, you distinctly hear a well marked *bruit de souffle*, coinciding with the first sound of the heart, and extending over the whole præcordial region; this sound is very loud near the apex of the organ, proving that it takes place when the ventricle contracts and sends the blood into the auricle. There is, then, hypertrophy of this cavity, with, at the same time, deficiency of the mitral valve, which causes the blood partially to reflow into the ventricle after its contraction. This is the chief cause of the dyspnœa we observe in this patient.

Independently of these organic lesions, the patient, for the last six weeks, has suffered from a catarrhal affection of the lung, which has very much increased the state of oppression in which you have seen her, and has, in part, contributed to the aggravation of the cardiac symptoms, and obliged her to lie by; for, probably, without this accidental pulmonary affection she would have been able for some time to come to attend to her occupations, even with her habitual dyspnœa. On auscultating the chest you hear a sibilant rhonchus extending over the whole of it, mingled on the left side with a little mucous rale. To the hypertrophy of the heart is therefore added a general bronchitis, dating about six weeks, and, in addition, an infiltration of the lower extremities, extending to the abdominal region.

This, however, is not all; the abdomen itself is infiltrated, and is at the same time meteoric; there is some serum, besides, in the cavity of the peritoneum. At first there is some difficulty in ascertaining the existence of ascites, on account of the anasarca of the abdominal integuments, which prevents your feeling the fluctuation. There is a means, however, of overcoming this obstacle. This consists in strongly compressing the abdominal parietes until the fingers are arrested, so to speak, by the sensation of a kind of tense pouch, which is the peritoneum; here you can easily distinguish the fluctuation of a liquid contained in the cavity of the abdomen. There sometimes exist, besides the dropsy, tumours in the abdomen, which, without this precaution, will be distinguished with difficulty. In such cases your exploration should be conducted with still greater attention. Pressure should be made firmly and quickly in such cases; by this means the stratum of fluid between the tumour and your hand is forced back, and you are able to feel the latter. With the aid of this means I have been enabled, in the present case, not only to determine the presence of ascites, but also the existence of a tumour, occupying the right side, and extending towards the left hypochondriac region. What is the nature of this tumour? Is it formed by the uterus anormally developed! I have made a vaginal examination and I have found the neck of the uterus soft, and half opened; but the organ itself is not enlarged, if you consider the recent period of delivery; it does not extend above the pubis; and the hypogastric region is, on percussion, sonorous. Besides, the principal seat of the tumour is the right side, although it extends slightly to the left. When you press upon this region, there is considerable resistance in the right hypochondriac and iliac regions, which continues obliquely across to the left side, where it is less. Is it the liver? Tumours of this organ are, in general, characterised by a kind of line or border, more or less defined and angular to the touch. This is a character almost constant. In the present case we have been unable to detect this important sign; or, if it exists, it is not well marked, and is masked probably by the œdema of the integuments. In spite, however, of the absence of this symptom, I believe that we have, in reality, an anormal development of the liver. Here, then, is another morbid complication, independent of those we have already discovered to exist.

There are, as you know, primitive, as well as consecutive lesions; the one primary, the other depending on some pre-existing morbid alteration. Now, disease of the liver may certainly be primitive, but most generally it is consecutive to some organic disease of the heart. Sometimes the hepatic symptoms lead to the discovery of the cardiac lesion before the latter had revealed itself by any direct sign. This is a very important means of diagnosis, and one that you should always remember in affections of this nature.

Such is the *ensemble* of the symptoms which I have been able to discover in this patient.

With regard to the heart, I have diagnosticated a hypertrophy of that organ, with valvular deficiency. It is possible that other lesions may exist, but our means of diagnosis of these diseases are yet too imperfect, in spite of all our recent discoveries, to permit us to push our diagnosis any further with certainty. The serum effused into the cavity of the abdomen is a phenomenon of the third order of symptoms, depending on the affection of the liver, in the same manner as the affection of that organ may be a consequence of that of the heart.

The prognosis is very grave. In addition to all the circumstances just enumerated, this patient was delivered at the Maternity by the forceps. After this difficult labour there was no swelling of the breasts; there was no well-marked milk fever; there was a very slight lochial discharge; and finally, the pulmonary symptoms appeared to aggravate all the other phenomena. This catarrhal affection resisted all the means employed to combat it, and the patient, suffering from bronchitis on her entrance into the Maternity, left it in the same state. All these circumstances are serious. We have, moreover, to fear the progress of the anasarca. You know that, in organic affections of the heart, the œdema appears and disappears ordinarily several times before a fatal termination. Whenever patients expose themselves to causes capable of increasing the intensity of the disease, as over fatigue, irregularities of living, moral emotions, the anasarca appears, and then, on the cessation of the cause, disappears to return again, and so on. But when this morbid phenomenon shows itself alone, without other cause than the principal affection, of which it is the ordinary consequence, it persists, without interruption, until the fatal issue of the disease. In our patient the hypertrophy appeared at the same time as the catarrhal affection, and has continued in spite of all treatment; there is, consequently, little hope of the disappearance of these phenomena, because we can in nowise count on the cure of the principal disease.

What can be done in an affection so complicated? Were the patient not in so feeble a condition, bloodletting would be indicated; but in her present state we do not dare to resort to it. We have employed the usual means in such circumstances, as the application of a large blister on the chest; the administration of Kermes Mineral and digitalis internally, to act on the circulatory system, and diminish its force; with purgatives of castor oil, with a drop of croton oil, to procure prompt and abundant evacuations, with mild mucilaginous drinks.

*Paris, Dec.* 1842.

